# Leptospirosis Prevalence in Patients with Initial Diagnosis of Dengue

**DOI:** 10.1155/2012/519701

**Published:** 2012-04-24

**Authors:** A. Dircio Montes Sergio, E. González Figueroa, Verdalet Guzmán María Saadia, Soler Huerta Elizabeth, Rivas Sánchez Beatriz, M. Altuzar Aguilar Víctor, J. Navarrete Espinosa

**Affiliations:** ^1^Coordinación de Vigilancia Epidemiológica y Apoyo en Contingencias, Unidad de Salud Pública, Instituto Mexicano del Seguro Social (IMSS), 03100, Mexico City, DF, Mexico; ^2^Coordinación de Salud Pública, Delegación Veracruz Norte, IMSS, 91810, Mexico City, DF, Mexico; ^3^Coordinación de Investigación en Salud, Delegación Veracruz Norte, IMSS, 91000, Mexico City, DF, Mexico; ^4^Clínica de Medicina Tropical, Unidad de Medicina Experimental, Facultad de Medicina, UNAM, 06700, Mexico City, DF, Mexico; ^5^Centro de Investigación en Micro y Nanotecnología, Universidad Veracruzana, 94294, Xalapa, VER, Mexico

## Abstract

*Objective*. To determine the prevalence of leptospirosis in patients from Veracruz with initial diagnosis of dengue and its association with risk factors. *Materials and Methods*. Transversal study in patients who sought medical attention under the suspicion of dengue. Backgrounds were researched and blood samples were drawn to determine dengue (NS1, RT-PCR) and leptospirosis (IFI). Simple frequencies, central tendency and dispersion measures, and prevalence and trust intervals at 95% (IC95%) were obtained. Prevalence reasons (RP) and IC_95%_ were obtained and a multivariate logistic model was applied, using SPSS V15. *Results*. 171 patients were included, 56% women (32 ± 14 years) and 44% men (32 ± 17 years). 48% of the cases (IC95% 40.5–55.4) was positive to dengue, with a cut point of 1 : 80, seroprevalence for leptospirosis was of 6% (IC_95%_ 2.7–10); 12% (IC95% 7–16.5) was positive to both pathologies and 34% was negative to both tests. Although the largest number of isolations corresponded to serotype 2, the four dengue virus serotypes were identified. In the bivariate analysis, overcrowding RP = 1.33, (IC = 0.46–3.5), bathing in rivers (RP = 1.31, IC = 0.13–7.4), and walking barefoot (RP = 1.39, IC = 0.58–3.3) were the variables associated with leptospirosis, although the relation was not statistically significant. *Conclusions*. Leptospirosis prevalence in subjects under suspicion of dengue fever is high, as well as the coincidence of both infections. The results show the coexistence of overlapped outbreaks of several diseases sharing the side of transmission. It is necessary the intentional search of other pathologies, such as influenza, rickettsiosis, and brucella, among others.

## 1. Introduction

Dengue is a viral disease caused by any of the known dengue viruses (Den1–Den4), and it is transmitted by the bite of a previously infected female *Aedes aegypti *mosquito. Literature describes several presentations of the disease, which go from asymptomatic to undifferentiated forms, dengue fever, hemorrhagic dengue fever [1–3]. Currently there exists a clinical classification of the disease that considers the nonsevere dengue and severe dengue [4].

During the last decades, the number of cases of dengue has notably increased, as well as the countries affected worldwide [5, 6]. In America, the dissemination and permanence of the vector, as well as the circulation of the four serotypes, have favored conditions of extensive and repeated exposure to the population, which has been reflected in an increase of hemorrhagic forms [7–9].

In Mexico, this pathology has increased and dispersed to all states with favorable ecological conditions for the reproduction of the vector [10–12] and even to areas not previously considered as favorable [13]. This fact is unveiled, when during 2009 more than 200,000 cases of dengue were notified in the country, and among them, 44,727 were DF and 11,398 HDF, approximately 40% of the registered cases corresponds to a population of beneficiaries of the IMSS [14].

Leptospirosis is a bacterial infection considered as a cosmopolitan zoonosis, present both in rural and urban areas where the environmental factors (weather, hydrographic distribution, urban infrastructure, etc.) are combined together with life styles (close contact with domestic animals and harmful fauna), promoting its development [15].

*Leptospira *is an extremely mobile bacterium which can survive in water, alkaline floor, and in humid or warm environments. An important characteristic of this bacterium is that it may remain for long periods of time in the kidney tubes in animals, being excreted by urine for many years without being the animal sick, thus becoming in asymptomatic carriers, aiding the animal to animal or animal to human transmission [16].

Humans become accidental guests by direct contact with urine or tissues from infected animals through injured skin, mainly in the feet or nasal or mouth mucosa or eyes or by indirect way (endemic outbreaks) by contact with stagnant water, dirt, or food contaminated with *Leptospira*.

After an incubation period of 1 or 2 weeks, the infection may follow two paths: asymptomatic and overcome it in most of the cases or the development of the disease, which may become acute or chronic; in its acute form, the symptoms may be undifferentiated or very mild or be present in a severe way, as a consequence of extensive vasculitis, known as Waill syndrom and that is characterized by liver and kidney damage or pulmonary hemorrhage commonly leading to death. The chronic form of the infection has been recognized until only few years ago and may present a large diversity of clinical manifestations, affecting different organs [15–19].

Some studies refer that approximately 500,000 are registered annually worldwide; in 2002, most incidents were reported in India (50.0/100,000 habitants) and Thailand (23.1/100,000 h). In America, the highest incidence was reported in Brazil (1.9/100,000 h) and the highest lethality rate was reported by Uruguay and Panama [20]. Studies are performed in Mexico reported antibody prevalence between 10 and 50% in different populations [21–24].

Since the last decades, it has been mentioned that climatic changes occurring in the planet could modify transmission patters of several infectious diseases [25, 26]. This fact becomes obvious when considering the reports of the transmission of new agents in our country [27] or overlapped outbreaks of different diseases, especially dengue and Leptospirosis, in Mexico and other countries [28, 29].

The relevance of this fact is that both infections is manifested by unspecific clinical symptoms, characterized by headaches, fever, myalgias, and arthralgias, which commonly leads to problems for a precise diagnosis and adequate treatment of the diseased, especially if they do not have antecedents of transmission of any of the diseases referred to, or it presents multiple forms of expression [16, 18, 19].

The coexistence of diverse pathogen agents that cause diseases with fever and hemorrhages in Mexico has forced the need to identify them and learn about the level of transmission of each disease, as well as the different clinical aspects that they share or make them different, to be able to offer a correct diagnosis and treatment.

To learn about the prevalence of leptospirosis in patients with diagnosis of probable dengue, as well as the factors associated with the infection and clinical behavior, a study was done in the population of Veracruz, state located in the Gulf of Mexico, characterized by tropical weather and abundant rains; currently it is considered as endemic for dengue and with increasing reports during the last years for leptospirosis cases [30]. The study was performed with the support of the Fondo de Investigación en Salud del IMSS (Health Research Fund from the IMSS) and the collaboration of the Centro de Investigación y Estudios Avanzados de el Instituto Politécnico Nacional (CINVESTAV-IPN) and the Departamento de Medicina Tropical de la Unidad de Medicina Experimental from the Medicine Faculty of the UNAM (FM-UNAM).

## 2. Materials and Methods

A transversal study was performed in the three Units of Family Medicine (UFM) (57, 61 y 68) and the Hospital General de Zona No.71 (General Zone Hospital-71 (GZH)), selected for their similarity in tending beneficiaries to the Social Security System (Instituto Mexicano del Seguro Social) in the port of Veracruz, for the type of services they grant, their structure, and the size of the attention units. Patients of all ages and genders who went for medical attention with an initial probable diagnosis of dengue fever (fever, headache, myalgia, arthralgia, and exanthema) were invited to participate, accepting their participation during the months of July through November 2009. A semistructured questionnaire was applied for their personal data and to research about the risk factors associated with dengue and leptospirosis: living conditions, overcrowding, protection measures against insects, contact with stagnant water, and direct contact with domestic animals.

Blood samples were drawn for dengue diagnosis (NS1 y RT-PCR: CINVESTAV-IPN) [31, 32] and leptospirosis (IFI, direct observation in dark field: FM-UNAM) [33, 34]. The tests for dengue target the identification and characterization of the dengue virus in the early stage of the disease; the tests for *Leptospira* were performed to measure the antibody titers against this agent to identify acute infection, as well as to demonstrate the presence of the bacterium in blood.

For the analysis of the information, simple frequencies, central tendency and dispersion measures, as well as prevalence (P) and trust intervals at 95% (IC95%) were obtained. As measure of association, the *χ* ^2^ test was used, and as measure of effect, prevalence ratios (PMR) were obtained [35] and IC95%. Finally, an unconditional multivariate logistic model adjusted to age was performed, using SPSS V15.

## 3. Results

From a total of 180 interviewed patients, 171 agreed to participate in the study, 28% from the FMU#57, 18% from the FMU#61, 37% from the FMU#68, and 17% from the GZH#71. The group was formed by 96 women and 75 men. The average age in males was 32 ± 17 years and 32 ± 14 years for women. The smallest group in number was the one conformed by children less than 5 years of age (3.5%).

The results of the diagnostic tests for *Leptospira* were demonstrated by means of direct observation in dark field, the presence of the agent in 168 patients (98%). However, indirect immunofluorescence demonstrated antibodies in 1 : 40 cut point in 84 cases (49%) and a 1 : 80 cut point in 31 cases (18%). These results demonstrated that 98% of the studied patients were infected with *Leptospira*, although only 18% presented an acute infection (1 : 80).

In relation to dengue, the four dengue virus serotypes were identified in the studied sample by RT-PCR. In total, 59 isolations were performed and the highest proportion was of DV type 2; serotype 1 in 5 cases, serotype 2 in 39 cases, serotype 3 in 8 cases, and serotype 4 in 7 cases (Table 1). In the rest of the cases, no presence of dengue virus was identified by this means; however, 43 of them were positive to identification to the NS1 antigen.

The combination of the results of the tests performed, identified 82 patients (48%) infected with the dengue virus, 11 (6.4%) infected with *Leptospira *(cut pint 1 : 80), and 20 cases (11.7%) infected with both agents; in 58 patients (33.9%) none of the agents were identified.

Regarding dengue diagnosis, 56 cases (32.7%) presented DF and 26 (15.2%) DHF. In combination with *Leptospira*, it was found that 15 (8.8%) had DF and *Leptospira* and 5 (2.9%) DHF and *Leptospira* (Table 2).

According to the unit of precedence, most dengue prevalence belonged to the GZH#71 (58.6%) and the FMU#57 (54.1%); for *Leptospira* was FMU#68 (34.9%) and FMU#57 (4.1%); for both infections, prevalence was higher in the FMU#57 (16.7%) and the FMU#68 (12.7%). All these units are located in the urban interface area of the port of Veracruz.

The largest proportion (26.9%) of the surveyed, referred being students, followed by those claiming administrative work, traders, and housewives (13.5, 12.9, and 11.7%, resp.).

From the studied patients, 76.6% said to have their own houses, 66.7% had concrete houses, 93.6% with tile or cement floor, and 88.3% had an English-type bathroom; also, 84.2% said living in an asphalted street. Considering living four or more persons per room, only 9.9% was overcrowded.

In the bivariate analysis, there were no differences found for leptospirosis according to gender and regarding the ages of the participants. The 35–39 years of age group, compared to the group of less than 5 years, showed a higher risk (RP = 2.33, I.C. 0.17–31.5). Likewise, those who said to live in overcrowded conditions presented a higher risk (RP = 1.33 IC_95%_ 0.46–3.5) when compared with those without this condition. Also, a higher risk was found in those patients who stated bathing in rivers, lagoons, and wells (RP = 1.31 IC95% 0.13–7.4) and in those who used to walk barefoot (RP = 1.39 IC95% 0.58–3.3); however, the associations were not statistically significant; this fact impeded the application of the multivariate logistic model.

For the analysis of clinic behavior, six groups were formed: DF, DHF, DF and *Leptospira*, DHF and *Leptospira*, leptospirosis, and other diagnosis. According to the presence of signs and cardinal dengue symptoms, fever was the symptom that was registered in the least proportion in all the group (82 to 96%), headache, myalgias, arthralgias, retroorbital pain, and exanthema were present in 98 to 100% in the studied patients, and there were no significant differences among the groups of patients.

The digestive signs and symptoms and alarming data were more common in the most severe cases; nausea and vomit were common in the DHF and DHF and *Leptospira*; diarrhea was present in a higher proportion in the DHF and *Leptospira*; abdominal pain was more common in the DHF and leptospirosis; hemorrhages and liquid permeation were mainly documented in the DHF, DHF and *Leptospira*, and the *Leptospira* groups (Table 3). No deaths were registered.

## 4. Discussion

The obtained results demonstrated the current problems represented by the diseases transmitted by vector and zoonosis in our country, considering the reported number of cases and leptospira, as well as the overlapping of these and other diseases in the different regions nation wide.

Traditionally, the Mexican Gulf area has been considered as a dengue endemic zone and it has recently been demonstrated the presence of other similar infectious diseases and a widening of the transmission curve dengue, with the subsequent increase in the number of registered cases and the increase in medical demand and costs.

Leptospirosis is a disease transmitted by contact with water contaminated with the urine of infected animals; the presence of intense rains which favor floods determine a higher exposure of the subjects to these conditions, especially in all those areas in the state of Veracruz where rain and contact with domestic animals and cattle are common denominators. This fact explains the high prevalence of infection by *Leptospira* that has been found. The present study demonstrated the presence of two different infectious agents in a group of feverish patients, becoming even more relevant after finding subjects infected with both agents.

The identification of the four dengue serotypes in the studied sample marks the endemic degree of the infection in the state. In the same way, the detection of *Leptospira* by means of direct observation of dark field in 98% of the studied cases confirms these facts. In this sense, it calls our attention that only 18% of these develops an acute state, belonging to infection with dengue and that 12% presented both infections, fact that not necessarily originated a more serious condition, contrary to what is expected.

The prevalence of acute leptospirosis that was found is similar to the one reported in other studies [28, 36] and is consistent with the risk factors that were found [22, 37]. This highlights the necessity for improving the environment, avoiding direct contact with potentially infected animals, and modifying the habits to decrease the exposure to this agent.

The clinical expression of leptospirosis includes an ample array of signs and symptoms, which may cause this disease to go unnoticed [18, 19]. The clinical behavior observed in the sample of studied patients does not represent statistical differences in relation to dengue, which implies problems at an operational level for its early diagnosis. For this reason, it is important the implementation of specific diagnostic tests that will allow detecting the infection in its early stages, thus, being able to offer an adequate treatment.

It is important to notice that, differently from other studies [36], the cases identified here, although acute, no data were found of jaundice, kidney, or liver failure or conjunctiva hemorrhage, which are commonly associated with the expression of the most serious cases of leptospirosis (Weill syndrome) [38]. Nevertheless, it is common that serious cases of leptospirosis are reported in the region.

According to our findings, at least a sixth part of the studied cases should have been treated with antibiotics to avoid complications that could lead to the death of the patients or a state of chronic carrier. On the other hand, the fact that in 34% of the feverish cases neither dengue nor *Leptospira* was identified indicates the presence of other infectious agents that are present at the same time and in the same space as dengue and leptospirosis, which increases the risk of mishandling the patients. All this emphasizes the importance of establishing an epidemiologic surveillance that will allow us the identification of all the possible infectious agents, according to each region. This fact is fundamental to be able to offer a population a good-quality medical attention, especially at this time when the potential risk of dissemination of other infectious diseases such as chikungunya, yellow fever [4], influenza, rickettsiosis, and others is present.

The results presented here offer important elements for the diagnosis of patients and decision-making in public health issues and force us to continue this line of research to broaden our knowledge of this and other infectious diseases.

## Figures and Tables

**Table 1:** Dengue virus identified in the groups.

Serotype	DF	HDF	DF and *Leptospira *	HDF and *Leptospira *	Total
1	1	0	3	1	5
2	5	1	24	9	39
3	0	0	5	3	8
4	3	0	3	1	7

Total	9	1	35	14	59

**Table 2:** Classification of the cases according to the etiologic agent.

Diagnosis	Cases	%
DF	56	33
DHF	26	15
DF and *Leptospira *	15	9
DHF and *Leptospira *	5	3
*Leptospira*	11	6
None	58	34

Total	171	100

**Table 3:** Other signs and symptoms according to diagnosis.

Signs and Symptoms	DF	DHF	DF and *Leptospira *	DHF and *Leptospira *	Leptospira	Other
Nausea	11	31	13	0	27	8
Vomit	14	34	13	20	0	16
Diarrhea	5	11	7	20	9	5
Abdominal pain	15	19	0	0	18	14
HEM. MANIF.	0	19	7	0	0	0
Permeation	7	58	7	20	20	9
